# The relationship between teacher care and Chinese college students’ physical education class learning satisfaction: the chain mediation effect of exercise enjoyment and exercise self-efficacy

**DOI:** 10.3389/fpsyg.2025.1691027

**Published:** 2025-12-15

**Authors:** Haixiong Chen, Jun Xiang

**Affiliations:** 1 Guangdong Vocational Institute of Sport, Guangzhou, China; 2 School of Physical Education and Health, Zhaoqing University, Zhaoqing, China

**Keywords:** teacher care, physical education class learning satisfaction, exercise enjoyment, exercise self-efficacy, college students

## Abstract

**Background:**

Teacher care is a key factor influencing college students’ satisfaction with physical education (PE) courses. Insufficient teacher care may diminish students’ learning motivation and exercise engagement, affecting their physical and mental well-being. Yet, the mechanisms linking teacher care to PE learning satisfaction remain unclear, particularly the roles of exercise enjoyment and exercise self-efficacy.

**Objective:**

To investigate the impact of teacher care on college students’ physical education class learning satisfaction in Chinese universities and to validate the mediating role of exercise enjoyment and exercise self-efficacy between the two.

**Methods:**

A clustered convenience sample of 1,198 college students from Chinese universities completed the Teacher Care Scale, Physical Education Class Satisfaction Scale, Exercise Enjoyment Scale, and Exercise Self-Efficacy Scale.

**Results:**

Teacher care was significantly and positively associated with students’ physical education (PE) class satisfaction (*B* = 0.136, *p* < 0.001). It also had significant positive associations with exercise enjoyment (*B* = 0.673, *p* < 0.001) and exercise self-efficacy (*B* = 0.176, *p* < 0.001). Exercise enjoyment was positively associated with exercise self-efficacy (*B* = 0.700, *p* < 0.001) and PE class satisfaction (*B* = 0.213, *p* < 0.001). Exercise self-efficacy was also significantly associated with PE class satisfaction (*B* = 0.268, *p* < 0.001). Further mediation analysis indicated that exercise enjoyment and exercise self-efficacy jointly mediated the relationship between teacher care and PE class satisfaction.

**Conclusion:**

This study enriches the theoretical understanding of motivational and affective mechanisms in physical education and offers practical implications for improving teaching quality and student well-being. Limitations include the cross-sectional design and self-report data, which restrict causal inference and generalizability. Future research could employ longitudinal or mixed-method approaches across more diverse educational contexts to validate and extend these findings.

## 1 Introduction

In recent years, student satisfaction with physical education (PE) courses has increasingly become a focal point of research in higher education. In recent years, student satisfaction with physical education (PE) courses has increasingly become a focal point of research in higher education. In China, the unique context of PE education makes this issue particularly salient. Under the ongoing Double Reduction policy, which emphasizes reducing academic burdens while improving physical and mental well-being, universities are required to strengthen PE curriculum reform and enhance students’ physical fitness levels (Liu and Wang, 2022). Moreover, national standards for college students’ physical fitness have placed additional pressure on universities to ensure both participation and performance. However, PE courses in many institutions still tend to prioritize “form over experience,” emphasizing technical skill drills and test preparation while neglecting students’ emotional engagement and learning enjoyment (He, 2012). These challenges may weaken students’ motivation and satisfaction with PE learning.

The PE course satisfaction typically refers to students’ overall subjective evaluation of the course content, teaching process, learning experience, and outcomes (Tao, 2025). Research indicates that high levels of satisfaction not only enhance students’ enthusiasm for participating in PE but also promote their physical and mental health and overall development (Cheon and Lim, 2020). Currently, scholars both domestically and internationally have extensively explored the influencing factors, measurement tools, and improvement pathways of physical education course learning satisfaction. Research has found that course content, teaching methods, and teacher-student interaction all significantly impact learning satisfaction (Miao et al., 2022). However, existing research has primarily focused on course design and teaching methods, with limited attention given to the role of social-psychological factors such as emotional support. Especially in the context of Chinese universities, there is still room for improvement in the overall level of learning satisfaction in physical education courses among college students, and the underlying mechanisms require further exploration. Therefore, systematically investigating the key factors influencing college students’ learning satisfaction in physical education courses and their underlying mechanisms holds significant theoretical and practical implications.

Teacher care, as an important form of educational emotional support, has garnered significant attention in higher education research in recent years. Teacher care typically refers to the respect, understanding, support, and attention teachers demonstrate toward students during the teaching process (O’Hare et al., 2020). In the context of physical education, however, teacher care takes on distinctive characteristics due to the interactive, skill-based, and physical nature of PE classes. It often manifests through teachers’ sensitivity to students’ physical conditions, timely encouragement during challenging tasks, guidance on injury prevention, and differentiated instruction that accommodates varying levels of athletic ability (Andersson et al., 2018; Rikard, 2009). Such care not only helps maintain students’ physical safety and confidence in movement learning but also fosters a psychologically supportive learning climate that promotes enjoyment and engagement (Sarwer et al., 2024). In the field of physical education, teacher care significantly influences students’ participation in physical activities, learning attitudes, and satisfaction (Zalech, 2021). However, empirical research on the relationship between teacher care and college students’ physical education class learning satisfaction remains limited, particularly lacking systematic analysis of its underlying mechanisms. Therefore, exploring how teacher care relates to students’ psychological experiences and their physical education class learning satisfaction has become an important research direction.

Additionally, exercise enjoyment and exercise self-efficacy, as important psychological variables influencing physical education learning experiences, may play a chain-mediated role between teacher care and physical education class learning satisfaction. Exercise enjoyment refers to the positive emotional experiences students gain from physical activities, while exercise self-efficacy reflects students’ confidence in their ability to participate in physical exercise (Wang et al., 2022). Research has found that teacher care can enhance students’ exercise enjoyment and exercise self-efficacy, thereby promoting their satisfaction with physical education learning (Sánchez-García et al., 2024). However, systematic empirical analyses of the specific pathways through which these two factors influence teacher care and physical education class learning satisfaction are lacking. Therefore, further exploring the relationships among teacher care, enjoyment of physical activity, exercise self-efficacy, and physical education class learning satisfaction not only enriches physical education theory but also provides new practical insights for enhancing college students’ physical education class learning experiences and satisfaction.

## 2 Literature review and research hypotheses

### 2.1 Teacher care and physical education class learning satisfaction

Teacher care typically refers to the respect, understanding, support, and attention that teachers demonstrate toward students during the teaching process. This positive teacher-student interaction can effectively enhance students’ learning motivation and sense of belonging (O’Hare et al., 2020; Sarwer et al., 2024). In the field of physical education, teacher care is not only reflected in the transmission of teaching content but also in attention to individual differences among students, emotional support, and diverse motivational approaches. Research indicates that teacher care significantly enhances students’ interest and participation in physical education courses, thereby influencing their overall learning experience (Guo et al., 2023). Furthermore, multiple surveys indicate that higher levels of teacher care are associated with greater student physical education class learning satisfaction (Leyton Román et al., 2019). For example, one study found a significant positive correlation between teacher care and learning satisfaction in physical education classes, meaning that teachers who provide more care and support during instruction can effectively enhance students’ learning satisfaction (Moral-Garcia et al., 2021). Additionally, research indicates that teacher care can indirectly promote positive evaluations and satisfaction with physical education courses by enhancing students’ self-confidence and sense of belonging (Granero-Gallegos et al., 2023). According to self-determination theory, teacher care serves as an important resource for exercise enjoyment, fulfilling students’ basic psychological needs and thereby enhancing their learning satisfaction (Leo et al., 2022). It is evident that teacher care not only directly influences students’ learning satisfaction but also indirectly exerts its effects through various psychological mechanisms. There is a high correlation between teacher care and learning satisfaction in physical education classes. Therefore, this study proposes Hypothesis 1: Teacher care is positively associated with college students’ learning satisfaction in physical education classes.

### 2.2 The mediating role of exercise enjoyment

Exercise enjoyment typically refers to the positive emotions and pleasant feelings experienced by individuals during physical activities (Wang et al., 2022). Research has found that teacher care can significantly enhance students’ levels of exercise enjoyment. Teachers who provide students with more attention, encouragement, and support in physical education classes help create a positive and relaxed classroom atmosphere, enabling students to derive greater enjoyment from physical activities (Leisterer and Jekauc, 2019). For example, a study found that teacher care is significantly positively correlated with college students’ enjoyment of physical activity, and that teachers’ emotional support and positive feedback can effectively stimulate students’ interest and enjoyment of physical activity (Sánchez-García et al., 2024). Additionally, scholars have pointed out that enjoyment of physical activity is not only influenced by teacher care but is also closely related to overall physical education class learning satisfaction. Higher levels of enjoyment of physical activity are associated with higher overall physical education class learning satisfaction. Pleasant physical activity experiences can enhance students’ positive evaluations of physical education courses, increase their willingness to participate, and boost their motivation for continued learning. Enjoyment of physical activity is an important predictive factor influencing overall physical education class learning satisfaction (Leyton Román et al., 2019). This finding indicates that exercise enjoyment plays an important role in enhancing physical education class learning satisfaction. Finally, according to self-determination theory, teacher care can satisfy students’ emotional and belonging needs, stimulate their intrinsic motivation, and thereby enhance exercise enjoyment. In turn, enjoyable exercise experiences further enhance students’ physical education class learning satisfaction (Trigueros et al., 2019). In summary, teacher care enhances students’ enjoyment of physical activity, which in turn promotes their physical education class learning satisfaction, and there is a significant mediating mechanism between the three. Therefore, this study proposes Hypothesis 2: Enjoyment of physical activity mediates the relationship between teacher care and college students’ physical education class learning satisfaction.

### 2.3 The mediating role of exercise self-efficacy

Exercise self-efficacy refers to an individual’s confidence and ability to successfully engage in physical exercise and complete related tasks (Tang et al., 2019). Research indicates that exercise self-efficacy is significantly positively correlated with students’ physical education class learning satisfaction. Students with higher levels of self-efficacy are more likely to exhibit positive participation attitudes and sustained learning motivation in physical education classes, thereby achieving higher levels of learning satisfaction (Morales-Sánchez et al., 2021). For example, a study also found that exercise self-efficacy not only enhances students’ interest in physical education classes but also increases their satisfaction with course content and teaching processes (Guo et al., 2020). This indicates that exercise self-efficacy is an important psychological mechanism influencing learning satisfaction in physical education classes. Secondly, scholars point out that teacher care, as a positive resource for exercise enjoyment, plays a crucial role in enhancing students’ exercise self-efficacy. Teachers providing students with more attention, encouragement, and guidance in physical education classes can help enhance students’ confidence in their exercise abilities (Siedentop and Van der Mars, 2022). Empirical research shows that there is a significant positive correlation between teachers’ caring behaviors and students’ exercise self-efficacy. Teachers’ caring behaviors can help students overcome difficulties and challenges in exercise by providing emotional support and positive feedback, thereby enhancing their exercise self-efficacy (Wu and Cai, 2025). In summary, teacher care promotes students’ physical education course learning satisfaction by enhancing their exercise self-efficacy, and there may be a significant mediating mechanism among the three. Therefore, this study proposes Hypothesis 3: Exercise self-efficacy mediates the relationship between teacher care and college students’ satisfaction with physical education courses.

### 2.4 The chain mediation effect of exercise enjoyment and exercise self-efficacy

Scholars have pointed out that exercise enjoyment not only directly enhances individuals’ positive emotional experiences but also promotes the continuity and proactivity of their physical activity by enhancing exercise self-efficacy (Wang et al., 2022). For example, research has found that the pleasurable experiences gained by college students in physical activities can significantly enhance their exercise self-efficacy, with stronger pleasure leading to higher confidence in their exercise abilities (Peng et al., 2025). According to self-determination theory, when students experience pleasure in physical activities, their intrinsic motivation is stimulated, thereby enhancing their positive evaluation of their abilities and self-confidence (Chen et al., 2020). It is evident that there is a close positive relationship between exercise enjoyment and exercise self-efficacy, with exercise enjoyment serving as an important psychological foundation for enhancing exercise self-efficacy.

Furthermore, according to the Context—Process—Outcome model (Roeser et al., 1996), there may be a chain-like mediating effect between teacher care, exercise enjoyment, exercise self-efficacy, and physical education class learning satisfaction. The Context—Process—Outcome model provides a systematic framework for understanding how school environments influence adolescents’ learning and development. In this model, context refers to the environment that affects students’ academic achievements and process refers to psychological functioning or condition (Xie et al., 2021). Recently, scholars have used this model to explain how environment factors affect adolescent development outcomes (e.g., Datu and Yuen, 2020; Xie et al., 2021). Within this framework, teacher care serves as a contextual factor, as it represents an interpersonal resource embedded in the social and emotional climate of PE classes. A caring teacher cultivates a supportive, respectful, and psychologically safe environment, which fosters students’ sense of belonging, security, and engagement in physical activities (Liang et al., 2025).

In contrast, exercise enjoyment and exercise self-efficacy function as process variables, reflecting students’ internal affective and motivational responses to contextual cues. Exercise enjoyment captures the pleasurable and satisfying emotions experienced during participation, which are strongly influenced by teachers’ supportive behaviors. When students derive enjoyment from exercise, they tend to perceive physical challenges as attainable and personally meaningful (Hagberg et al., 2009), thereby reinforcing their exercise self-efficacy beliefs—the confidence in their ability to perform exercise tasks successfully. This process is further supported by the Broaden-and-Build Theory of Positive Emotions, which posits that positive emotions such as joy and enjoyment expand individuals’ thought–action repertoires and help them build enduring personal resources, including confidence and resilience (Fredrickson, 2004). Consistent with this reasoning, empirical studies have shown that teacher care enhances students’ exercise enjoyment, which in turn strengthens their exercise self-efficacy and ultimately improves satisfaction with PE classes (Sánchez-García et al., 2024; Guo et al., 2020). Therefore, this study proposes Hypothesis 4: Exercise enjoyment and exercise self-efficacy have a chain-like mediating effect between teacher care and physical education class learning satisfaction.

**Figure 1:**
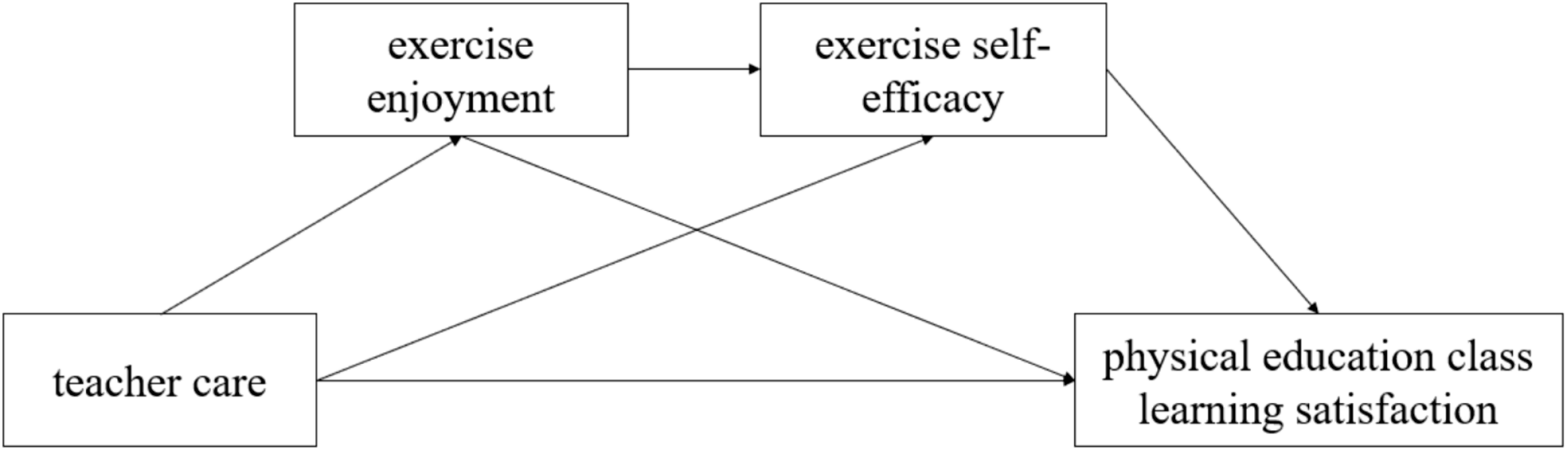
Research framework diagram.

In summary, scholars have identified the association between teacher care and college students’ satisfaction with physical education courses, emphasizing the mediating and chain mediating roles of exercise enjoyment and exercise self-efficacy in this relationship. These research findings provide the theoretical basis for the hypotheses of this study. Therefore, the research framework of this study is established as follows (Figure 1): (1) To examine the predictive role of teacher care on college students’ satisfaction with physical education courses; (2) To examine the mediating role of exercise enjoyment in the relationship between teacher care and college students’ satisfaction with physical education courses; (3) To examine the mediating role of exercise self-efficacy in the relationship between teacher care and college students’ satisfaction with physical education courses; (4) To test the chain mediating role of exercise enjoyment and exercise self-efficacy in the relationship between teacher care and college students’ satisfaction with physical education courses.

## 3 Research participants and methods

### 3.1 Research participants

Data were collected through a clustered convenience sampling approach rather than a purely random sample. To enhance representativeness, the sampling frame included universities from five major regions of China—East, Central, South, Southwest, and Northwest China—reflecting an approximate regional distribution of 42% Eastern, 27% Central, and 31% Western regions. The sample further balanced urban diversity, including about 38% participants from first-tier cities (e.g., Beijing, Shanghai, and Guangzhou) and 62% from non-first-tier cities. Regarding institution type, the sample comprised approximately 56% comprehensive universities, 28% normal universities, and 16% physical education universities. Stratification by gender (53% male, 47% female) and academic year was applied within institutions to ensure demographic balance.

When determining the sample size and collecting data for this study, we combined psychological and educational experience with the analytical methods used to preliminarily set the number of participants for each observed variable at 10–20, with a theoretical minimum sample size of 400–600 people. To enhance statistical efficiency and the generalizability of results, we referenced Cohen’s recommendations and utilized G*Power software for calculations. Using a medium effect size (d = 0.5), statistical power (power = 0.8), and significance level (*α* = 0.05) as standards (Cohen, 1992), the optimal sample size was determined to be approximately 800–1,000 participants. Considering the possibility of invalid questionnaires and missing data in the actual survey, the final number of questionnaires distributed was set at 1,270. Second, all tests were administered as on-site group tests in a physical education classroom setting. Prior to data collection, ethical approval was obtained, and written informed consent was secured from all participants. The consent procedure, approved by the ethics committee, clarified that participation was voluntary and part of a class-based activity. All sessions were conducted by professionally trained physical education teachers and psychology students using standardized instructions to ensure participants fully understood the questionnaire content. Strict inclusion criteria were applied: participants were limited to currently enrolled college students who attended physical education classes at least once per week. All questionnaires were completed within 10 min. After collection, researchers screened the data, excluding samples with missing data, response inconsistencies, or failure to meet the inclusion criteria. Ultimately, 1,200 valid questionnaires were obtained, with an effective response rate of 94.33%. Among these, 72 were excluded due to missing data for primary variables or failure to meet inclusion criteria. Third, participants were aged between 17 and 23 years old, with an average age of 20.03 years (standard deviation of 1.48). There were 615 males (51.25%) and 585 females (48.75%). There were no significant differences in scores across major variables based on gender or age, indicating a reasonable and representative sample structure. During the study, background variables such as gender and age were effectively controlled.

### 3.2 Research methods

#### 3.2.1 Psychological measurement methods

##### 3.2.1.1 Teacher Care Scale

The Teacher Care Questionnaire developed by Chen et al. (2019) was used to assess college students’ evaluations of teacher care in a Chinese cultural context. The scale consists of 23 items across five dimensions: instructional adaptation (e.g., “If an exercise is too difficult for me, the teacher will provide me with an alternative exercise or simplify the exercise), Classroom Atmosphere (e.g., the teacher uses humorous explanations to stimulate our interest in physical education), Emotional Perception (e.g., the teacher is friendly toward us in class), Physical Health (e.g., if I am unwell or ill, the teacher will continue to monitor my recovery), and Teacher-Student Relationship (e.g., my relationship with the physical education teacher is both teacher-student and friend). This scale uses a 5-point Likert scale (5 = strongly agree, 4 = somewhat agree, 3 = unsure, 2 = somewhat disagree, 1 = strongly disagree). The total score is calculated by summing the scores, with higher scores indicating a better evaluation of the physical education teacher’s care. In previous studies, the Physical Education Teacher Care Scale has been used to measure the level of teacher care among Chinese university students, demonstrating good reliability and validity (Chen et al., 2019). In this study, the reliability coefficients were Cronbach’s *α* = 0.538 and McDonald’s *ω* = 0.639 (95% CI [0.60, 0.67]). The CFA results indicated an acceptable fit (*χ*^2^/df = 1.982, CFI = 0.956, TLI = 0.940, RMSEA = 0.054 [90% CI [0.039, 0.068]], SRMR = 0.042), with standardized factor loadings ranging from 0.52 to 0.63. Multi-group measurement invariance analysis across gender (Configural → Metric: ΔCFI = 0.006; Metric → Scalar: ΔCFI = 0.004) showed acceptable invariance across gender. Therefore, this scale demonstrated acceptable reliability and good construct validity in this study.

##### 3.2.1.2 Exercise Enjoyment Scale

The Exercise Enjoyment Questionnaire, originally developed by Kendzierski and DeCarlo (1991) and revised by Motl et al. (2001), was used to measure the level of enjoyment individuals experience during any physical activity (Kendzierski and DeCarlo, 1991; Motl et al., 2001). It was subsequently translated and adapted by Chinese scholar Zhiqi-Sun to assess individuals’ enjoyment of physical activities within a Chinese cultural context (Sun, 2014). The scale consists of 16 items (e.g., Item 9: “I feel good physically when engaging in the activity”; Item 15: “Physical activities make me feel good”). The scale uses a 5-point Likert scale (5 = strongly agree, 4 = somewhat agree, 3 = unsure, 2 = somewhat disagree, 1 = strongly disagree). The total score is calculated by summing the scores, with higher scores indicating stronger enjoyment of physical activity. In previous studies, the Physical Activity Enjoyment Scale has been used to measure Chinese students’ enjoyment of physical activity, demonstrating good reliability and validity (He, 2023). In this study, Cronbach’s *α* = 0.684 and McDonald’s *ω* = 0.721 (95% CI [0.69, 0.75]). The CFA results showed a good model fit (*χ*^2^/df = 2.147, CFI = 0.948, TLI = 0.931, RMSEA = 0.049 [90% CI [0.034, 0.062]], SRMR = 0.038). The standardized factor loadings ranged from 0.65 to 0.78. Measurement invariance across gender was supported (Configural → Metric: ΔCFI = 0.008; Metric → Scalar: ΔCFI = 0.005), indicating equivalent measurement structure, loadings, and intercepts across gender. Overall, the scale exhibited acceptable reliability and satisfactory construct validity.

##### 3.2.1.3 Exercise Self-Efficacy Scale

The Exercise Self-Efficacy Questionnaire developed by Saunders et al. (1997) was used to assess individuals’ levels of exercise self-efficacy. The scale consists of 12 items (e.g., Item 1: “Most days of the week, I exercise”; Item 5: “I can ask my best friend to exercise with me most days of the week”). The scale uses a 5-point Likert scale (1 indicates strongly disagree, 2 indicates somewhat disagree, 3 indicates unsure, 4 indicates somewhat agree, and 5 indicates strongly agree). The total score is calculated by summing the scores, with higher scores indicating better exercise self-efficacy. In previous studies, the Exercise Self-Efficacy Scale has been used to measure Chinese students’ exercise self-efficacy levels, demonstrating good reliability and validity (Chen et al., 2019). In this study, Cronbach’s *α* = 0.680 and McDonald’s *ω* = 0.709 (95% CI [0.67, 0.74]). CFA results indicated a satisfactory model fit (*χ*^2^/df = 2.328, CFI = 0.941, TLI = 0.926, RMSEA = 0.052 [90% CI [0.038, 0.065]], SRMR = 0.046), with standardized factor loadings between 0.64 and 0.72 (all *p*s < 0.001). Gender invariance testing (Configural → Metric: ΔCFI = 0.009; Metric → Scalar: ΔCFI = 0.006) confirmed that the factor structure was invariant across gender. These results suggest acceptable reliability and good construct validity.

##### 3.2.1.4 Physical Education Class Learning Satisfaction Scale

The Physical Education Class Learning Satisfaction Questionnaire developed by Cunningham (2007) was adapted and translated by Chinese scholar Qing-Shi into the Physical Education Class Learning Satisfaction Scale, which was used to assess students’ levels of physical education class learning satisfaction in a Chinese cultural context (Shi, 2010). The scale consists of 27 items across five dimensions: teaching atmosphere and content (e.g., teaching organization and learning atmosphere are good), teacher teaching ability (e.g., teachers can accurately grasp teaching priorities), peer relationships (e.g., students can receive encouragement and assistance from peers), facilities and equipment (e.g., the quantity of sports facilities and equipment is adequate), and performance evaluation (e.g., the grading criteria for exams are reasonable). The scale uses a 5-point Likert scoring method (1 = completely disagree, 2 = somewhat disagree, 3 = neutral, 4 = agree, 5 = strongly agree). The total score is calculated by summing the scores, with higher scores indicating higher physical education class learning satisfaction. In previous studies, the Physical Education Class Learning Satisfaction Scale has been used to measure Chinese students’ physical education class learning satisfaction, demonstrating good reliability and validity (Shi, 2012). In this study, Cronbach’s *α* = 0.610 and McDonald’s *ω* = 0.673 (95% CI [0.64, 0.70]). The CFA model fit indices were *χ*^2^/df = 2.085, CFI = 0.945, TLI = 0.929, RMSEA = 0.055 (90% CI [0.041, 0.068]), SRMR = 0.040. The standardized factor loadings ranged from 0.58 to 0.66 (all *p*s < 0.001). Gender invariance analysis (Configural → Metric: ΔCFI = 0.007; Metric → Scalar: ΔCFI = 0.003) confirmed stable measurement properties across gender. These results indicate acceptable internal consistency and good construct validity of the scale in the present study.

#### 3.2.2 Mathematical statistics method

In the data statistical analysis phase of this study, SPSS 21.0 software and Amos 26.0 were used for statistical analysis. Specifically, first, using SPSS 21.0 software, descriptive statistical analysis was conducted on the demographic information data collected, as well as the test data related to coaches’ leadership behavior, exercise enjoyment, exercise self-efficacy, and physical education class learning satisfaction, to present the basic characteristics and distribution of each variable. Additionally, difference tests were conducted, with a significance level set at *p* < 0.05, to determine whether there were significant differences between different groups in these variables. Second, to explore the intrinsic relationships among the four variables of coaches’ leadership behavior, enjoyment of sports, exercise self-efficacy, and physical education class learning satisfaction among college student athletes, this study used Amos 26 to test the mediating model between autonomous fitness behavior and positive mental health literacy. Indirect effects were determined through the bias-corrected bootstrapping procedure with 5,000 bootstrap samples. A mediating effect was deemed significant if the 95% confidence interval (CI) of effect sizes did not span zero. The model’s goodness of fit was assessed based on the following criteria: chi-square ratio to degrees of freedom (*χ*^2^/df) < 5.0, root mean square error of approximation (RMSEA) < 0.08, Tucker-Lewis index (TLI) > 0.90, Standardized Root Mean Square Residual (SRMR) < 0.08, and comparative fit index (CFI) > 0.90 (West et al., 2023). A two-tailed *p*-value < 0.05 was regarded as statistically significant.

## 4 Results

### 4.1 Descriptive statistical analysis of teacher care, physical education class learning satisfaction, exercise enjoyment, and exercise self-efficacy

Table 1 shows that physical education class learning satisfaction, exercise enjoyment, and exercise self-efficacy were statistically significant in gender difference analysis (*p* < 0.05), while teacher care was not statistically significant (*p* > 0.05). all four variables showed no statistically significant differences in age-based analysis (*p* > 0.05); Male students had higher average scores than female students in the three variables of physical education class learning satisfaction, exercise enjoyment, and exercise self-efficacy, while the teacher care variable was lower than that of female students. Additionally, the four variables exhibited certain patterns across different statistical measures, which helps this study further understand the extent of influence and interrelationships between teacher care, exercise enjoyment, and exercise self-efficacy on physical education class learning satisfaction (Table 2).

**Table 1:** Gender differences in teacher care, physical education class satisfaction, exercise enjoyment, and exercise self-efficacy.

Variable	Gender	Number (%)	*M*	*SD*	*t*	*p*
Teacher care	Male	615(51.25%)	80.63	10.10	−0.197	0.844
	Female	585(48.75%)	80.74	10.26		
	Total	1,200	80.68	10.18		
Physical education class learning satisfaction	Male	615(51.25%)	96.79	11.81	4.009^***^	<0.001
	Female	585(48.75%)	94.02	12.08		
	Total	1,200	95.44	12.02		
Exercise enjoyment	Male	615(51.25%)	58.22	7.88	5.110^***^	<0.001
	Female	585(48.75%)	55.88	7.99		
	Total	1,200	57.08	8.02		
Exercise self-efficacy	Male	615(51.25%)	33.16	4.65	4.636^***^	<0.001
	Female	585(48.75%)	31.88	4.96		
	Total	1,200	32.54	4.84		

^*^p < 0.05, ^**^p < 0.01, ^***^p < 0.001.

**Table 2:** Age differences in teacher care, physical education class satisfaction, exercise enjoyment, and exercise self-efficacy.

Variable	Age	Number (%)	*M*	*SD*	*F*	*p*
Teacher care	17	53(4.42%)	79.79	9.88	0.433	0.857
	18	127(10.58%)	81.01	10.61		
	19	265(22.08%)	80.59	10.46		
	20	305(25.42%)	80.21	9.99		
	21	252(21.00%)	81.35	10.15		
	22	133(11.08%)	80.43	10.43		
	23	65(5.42%)	81.28	8.95		
	Total	1,200	80.68	10.18		
Physical education class learning satisfaction	17	53(4.42%)	95.55	9.91	0.109	0.995
	18	127(10.58%)	95.08	13.71		
	19	265(22.08%)	95.91	12.14		
	20	305(25.42%)	95.27	11.45		
	21	252(21.00%)	95.33	11.92		
	22	133(11.08%)	95.56	12.29		
	23	65(5.42%)	95.15	12.57		
	Total	1,200	95.44	12.02		
Exercise enjoyment	17	53(4.42%)	58.57	7.86	1.296	0.256
	18	127(10.58%)	57.23	9.02		
	19	265(22.08%)	57.32	7.74		
	20	305(25.42%)	56.41	8.10		
	21	252(21.00%)	57.43	7.74		
	22	133(11.08%)	57.53	8.21		
	23	65(5.42%)	55.48	7.28		
	Total	1,200	57.08	8.02		
Exercise self-efficacy	17	53(4.42%)	31.75	5.29	1.679	0.123
	18	127(10.58%)	31.50	5.29		
	19	265(22.08%)	32.88	4.68		
	20	305(25.42%)	32.50	4.76		
	21	252(21.00%)	32.63	4.64		
	22	133(11.08%)	32.76	5.04		
	23	65(5.42%)	33.17	4.79		
	Total	1,200	32.54	4.84		

^*^p < 0.05, ^**^p < 0.01, ^***^p < 0.001.

### 4.2 Common method bias test

To examine potential common method bias, an unmeasured latent method factor (ULMC) was added to the baseline CFA model. The method factor was specified to load on all observed indicators, while the original latent structure was retained to distinguish substantive factors from the method factor (Ding et al., 2023).

Model fit indices indicated that the inclusion of the method factor resulted in only negligible improvement compared with the baseline model (*χ*^2^/df = 2.432, CFI = 0.943, TLI = 0.932, RMSEA = 0.056 (90% CI [0.045, 0.067]), SRMR = 0.045) and the model with method factor: *χ*^2^/df = 2.398, CFI = 0.945, TLI = 0.933, RMSEA = 0.055 (90% CI [0.044, 0.066]), SRMR = 0.043. The change in CFI (ΔCFI = 0.002) did not reach the conventional threshold for meaningful improvement (ΔCFI ≥ 0.01). Standardized loadings of the method factor ranged from 0.03 to 0.14, all below 0.15, and most were non-significant, suggesting minimal influence of common method variance. Furthermore, the factor loadings remained stable across models (all changes < 0.05), indicating that the inclusion of the method factor did not affect the underlying measurement structure.

### 4.3 Correlation analysis of teacher care, physical education class learning satisfaction, exercise enjoyment, and exercise self-efficacy

As shown in Table 3, teacher care is significantly positively correlated with physical education class learning satisfaction, exercise enjoyment, and exercise self-efficacy; physical education class learning satisfaction is significantly positively correlated with exercise enjoyment and physical education class learning satisfaction; and exercise enjoyment is significantly positively correlated with exercise self-efficacy. It is evident that the research variables (teacher care, physical education class learning satisfaction, exercise enjoyment, and exercise self-efficacy) are significantly correlated. The relationships between these variables support subsequent hypothesis testing and provide a solid foundation for the mediation effect analysis in this study.

**Table 3:** Correlation analysis statistics for teacher care, physical education class learning satisfaction, exercise enjoyment, and exercise self-efficacy.

Variable	M ± SD	Teacher care	Physical education class learning satisfaction	Exercise enjoyment	Exercise self-efficacy
Teacher care	80.68 ± 10.18	1			
Physical education class learning satisfaction	95.44 ± 12.02	0.450**	1		
Exercise enjoyment	57.08 ± 8.02	0.594**	0.559**	1	
Exercise self-efficacy	32.54 ± 4.84	0.472**	0.585**	0.655**	1

*N* = 1,200. **p < 0.01. Same below.

### 4.4 Testing the mediating effect of teacher care on physical education class learning satisfaction

**Figure 2:**
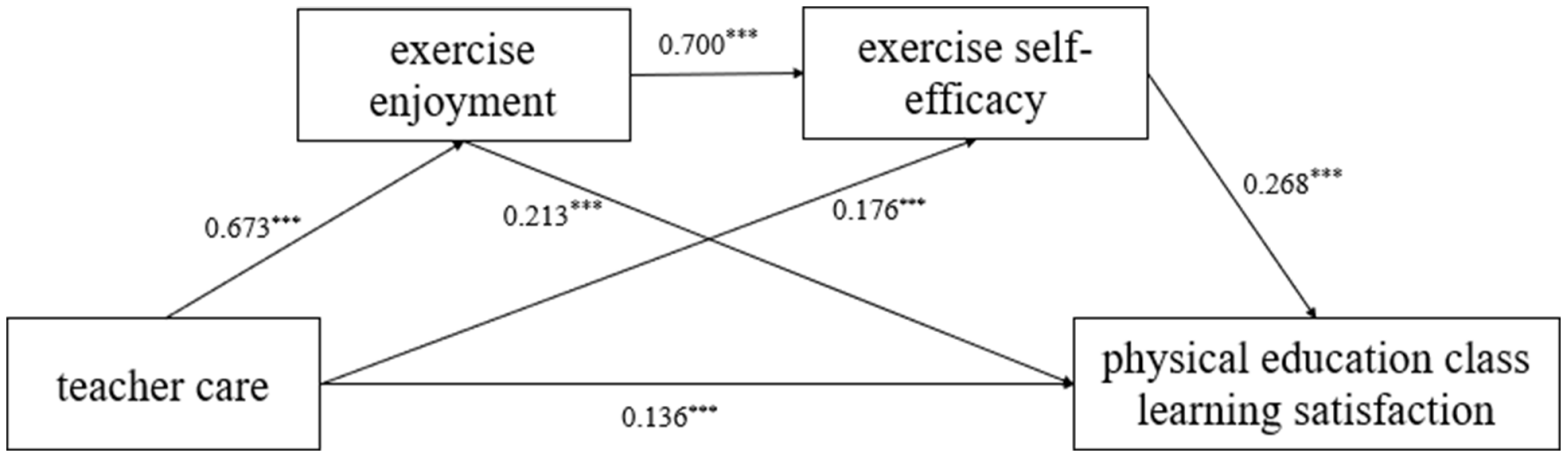
Chain mediation model of exercise enjoyment and exercise self-efficacy between teacher care and physical education class learning satisfaction. *p < 0.05, **p < 0.01, ***p < 0.001.

With teacher care as the independent variable, with exercise enjoyment and exercise self-efficacy as mediating variables, physical education class learning satisfaction as the dependent variable, and gender and age as covariate variables, we conducted a chained mediation analysis using structural equation modeling (SEM) in AMOS. The confirmatory analysis with the maximum likelihood method shows a good fit of the measurement model: CFI = 0.965, TLI = 0.958, RMSEA = 0.041, SRMR = 0.038. As shown in Figure 2, the results indicated that teacher care significantly and positively associated with exercise enjoyment (*B* = 0.673, SE = 0.026, *p* < 0.001) and exercise self-efficacy (*B* = 0.176, SE = 0.037, *p* < 0.001). Exercise enjoyment significantly positively associated with exercise self-efficacy (*B* = 0.700, SE = 0.032, *p* < 0.001) and physical education class learning satisfaction (*B* = 0.213, SE = 0.029, *p* < 0.001). Both exercise self-efficacy (*B* = 0.268, SE = 0.022, *p* < 0.001) and teacher care (*B* = 0.136, SE = 0.028, *p* < 0.001) were also significant positively associated with physical education class learning satisfaction. These results validate the research hypothesis H1.

After conducting standardized effect size analysis and significance tests on the pathways through which teacher care influences college students’ satisfaction with physical education courses (Table 4), the Bootstrap 95% confidence interval for the total indirect effect of teacher care on college students’ physical education class learning satisfaction does not include 0, indicating that “exercise enjoyment” and “exercise self-efficacy” have a significant mediating effect between teacher care and physical education class learning satisfaction (direct effect accounts for 31.20%, indirect effect accounts for 68.80%). This mediating effect consists of three indirect effects: (1) The indirect effect generated by the path from “teacher care” to “exercise enjoyment” to “physical education class learning satisfaction,” whose Bootstrap 95% confidence interval does not include 0, indicates that exercise enjoyment plays a significant mediating role between teacher care and learning satisfaction in physical education classes (standardized value = 0.118, accounting for 27.00% of the total effect), This result validates Hypothesis H2; (2) the indirect effect generated by the path from “teacher care” to “exercise self-efficacy” to “satisfaction with physical education class learning,” whose Bootstrap 95% confidence interval does not include 0, indicating that exercise self-efficacy plays a significant mediating role between teacher care and satisfaction with physical education class learning (standardized value = 0.057, accounting for 13.00% of the total effect), this result validates Hypothesis H3; (3) The indirect effect generated by the path from “teacher care” to “exercise enjoyment” to “exercise self-efficacy” to “physical education class learning satisfaction” has a Bootstrap 95% confidence interval that does not include 0, indicating that exercise enjoyment and exercise self-efficacy play a significant chained mediating role between teacher care and physical education class learning satisfaction (standardized value = 0.126, accounting for 28.80% of the total effect). This result validates hypothesis H4.

**Table 4:** Testing the chain mediation effect of exercise enjoyment and exercise self-efficacy between teacher care and physical education class learning satisfaction.

Effect type	Effect value	Boot SE	Bootstrap 95% CI		Effect percentage
			Lower limit	Upper limit	
Total effect	0.438	0.034	0.371	0.504	100%
Direct effect	0.136	0.028	0.081	0.193	31.20%
Teacher care – exercise enjoyment – physical education class learning satisfaction	0.118	0.023	0.074	0.164	27.00%
Teacher care – exercise self-efficacy – physical education class learning satisfaction	0.057	0.007	0.044	0.071	13.00%
Teacher care – exercise enjoyment – sexercise self-efficacy – physical education class learning satisfaction	0.126	0.012	0.103	0.152	28.80%

## 5 Discussion

### 5.1 Teacher care and physical education class learning satisfaction

The results of this study show that there is a significant positive correlation between teacher care and college students’ physical education class learning satisfaction, and that teacher care has a direct positive impact on physical education class learning satisfaction. This is consistent with the results of previous studies. The underlying reason is that teacher care, as a form of positive educational emotional support, can meet students’ emotional needs during physical education learning, enhance their sense of belonging and security, and thereby improve their overall satisfaction with physical education courses (Leo et al., 2022). Research has found that the correlation coefficient between teacher care and satisfaction with physical education courses is relatively high. The respect, understanding, and support demonstrated by teachers in physical education courses can effectively enhance students’ learning experiences and satisfaction (Moral-Garcia et al., 2021). Teachers’ positive feedback and emotional care can stimulate students’ learning interest and participation enthusiasm, further promoting their positive evaluation of physical education courses (Guo et al., 2023). Secondly, social support theory posits that teacher care, as an important social support resource, can meet students’ basic psychological needs, thereby enhancing their learning satisfaction (Kassis et al., 2019). Self-determination theory emphasizes that teacher care can stimulate students’ intrinsic motivation, enhance their autonomy and learning motivation, thereby improving their satisfaction with physical education courses (Leo et al., 2022; Chen et al., 2020). It is evident that teachers’ emotional support and positive feedback can significantly improve students’ learning experience and satisfaction with physical education courses, and teacher care plays a key role in improving college students’ satisfaction with physical education courses. Further analysis also reveals that teacher care not only directly influences physical education class learning satisfaction but may also indirectly exert effects through various psychological mechanisms, such as enhancing students’ self-confidence, sense of belonging, and learning motivation. Research indicates that teacher care can indirectly promote improvements in physical education class learning satisfaction by enhancing students’ exercise self-efficacy and enjoyment of physical activity (Morales-Sánchez et al., 2021). This aligns with the findings of this study, demonstrating that teacher care has multidimensional and profound positive impacts in physical education. Therefore, enhancing teacher care not only helps optimize the physical education classroom atmosphere but also effectively promotes the improvement of college students’ satisfaction with physical education courses, providing important theoretical foundations and practical guidance for physical education reform and talent cultivation in higher education institutions.

### 5.2 The mediating role of exercise enjoyment

This study found that exercise enjoyment had a significant mediating role between teacher care and physical education class learning satisfaction, further validating Hypothesis 2. This is consistent with the results of previous studies. The reason for this is that, first, there is a close positive correlation between exercise enjoyment and physical education class learning satisfaction. Enjoyment of physical activity refers to the positive emotions and pleasant feelings experienced by students during physical activities. This enjoyable experience significantly enhances students’ overall evaluation and satisfaction with physical education courses (Alcaraz-Muñoz et al., 2020). Research has shown that enjoyment of physical activity is an important psychological mechanism influencing physical education class learning satisfaction. The higher the enjoyment of physical activity, the higher the students’ physical education class learning satisfaction. Enjoyable physical activity experiences can enhance students’ willingness to participate and their motivation to continue learning (Zheng et al., 2023). Additionally, there is a significant positive relationship between teacher care and exercise enjoyment. When teachers provide students with more attention, encouragement, and support during physical education classes, it helps create a positive and relaxed classroom atmosphere, enabling students to derive greater enjoyment from physical activities (Leisterer and Jekauc, 2019). Research has found that teacher care is significantly positively correlated with college students’ enjoyment of physical activity. Teachers’ emotional support and positive feedback can effectively stimulate students’ interest in physical activity and their enjoyment of it (Trigueros et al., 2019). Self-determination theory also suggests that teacher care can satisfy students’ emotional and belonging needs, stimulate their intrinsic motivation, and thereby enhance their enjoyment of physical activity (White et al., 2021). Finally, teacher care enhances students’ enjoyment of physical activity, which in turn promotes their physical education class learning satisfaction, with a significant mediating mechanism among the three. Research shows that teacher care not only directly influences physical education class learning satisfaction but also indirectly affects learning satisfaction through the mediating pathway of “enjoyment of physical activity” (Zhang et al., 2025; Sánchez-García et al., 2024). This mechanism suggests that teacher care creates a positive learning environment for students, enhances their enjoyment of physical activity, and that this enjoyable experience further increases their physical education class learning satisfaction.

### 5.3 The mediating role of exercise self-efficacy

This study found that exercise self-efficacy plays a significant mediating role between teacher care and physical education class learning satisfaction, consistent with previous research findings. First, scholars argue that there is a close positive correlation between exercise self-efficacy and physical education class learning satisfaction (Wang et al., 2022). Exercise self-efficacy refers to students’ confidence and ability to successfully engage in physical exercise and complete related tasks. Research indicates that students with higher exercise self-efficacy exhibit stronger participation intentions and sustained learning motivation in physical education classes, thereby achieving higher learning satisfaction. Developing exercise self-efficacy not only enhances students’ interest in physical education classes but also increases their satisfaction with course content and teaching processes (Sánchez-García et al., 2024). It is evident that exercise self-efficacy is an important psychological mechanism influencing learning satisfaction in physical education classes. Secondly, teacher care plays a significant role in enhancing exercise self-efficacy. Teachers who provide students with more attention, encouragement, and guidance in physical education classes help enhance students’ confidence in their exercise abilities (Siedentop and Van der Mars, 2022). According to social cognitive theory, external support and positive feedback are important pathways for enhancing individuals’ exercise self-efficacy (Bandura, 2023). Research has found a significant positive correlation between teachers’ caring behaviors and students’ exercise self-efficacy. Teachers’ caring behaviors can help students overcome difficulties and challenges in exercise by providing emotional support and positive feedback, thereby enhancing their exercise self-efficacy. The more teachers care for and support students, the stronger their exercise self-efficacy becomes (Lam, 2019; Wu and Cai, 2025). In summary, teacher care enhances students’ exercise self-efficacy, thereby promoting their physical education class learning satisfaction, and there is a significant mediating mechanism among the three. Teacher care not only directly influences physical education class learning satisfaction but also indirectly affects learning satisfaction through the mediating path of “exercise self-efficacy.” This study indicates that teacher care provides students with positive social support, enhances their exercise self-efficacy, and high exercise self-efficacy further improves students’ physical education class learning satisfaction.

### 5.4 The chain mediation effect of exercise enjoyment and exercise self-efficacy

This study further found that exercise enjoyment and exercise self-efficacy constitute a chain mediation effect between teacher care and physical education class learning satisfaction, which is consistent with previous research findings. First, there is a close positive relationship between exercise enjoyment and exercise self-efficacy. According to self-determination theory, when students experience pleasure in physical activities, their intrinsic motivation is stimulated, thereby enhancing their confidence in their exercise abilities and exercise self-efficacy (Vasconcellos et al., 2020). Scholars have pointed out that the pleasurable experiences gained by college students in physical activities can significantly enhance their exercise self-efficacy; the stronger the pleasure, the higher the students’ confidence in their exercise abilities (Vasconcellos et al., 2020). This suggests that exercise enjoyment not only directly enhances individuals’ positive emotional experiences but also promotes the continuity and proactivity of physical activity by enhancing exercise self-efficacy. Secondly, exercise enjoyment plays an important mediating role between teacher care and physical education class learning satisfaction. This is because teacher care can enhance students’ physical education class learning satisfaction by increasing their exercise enjoyment (Moral-Garcia et al., 2021). Teachers providing students with more attention, encouragement, and support in physical education classes helps create a positive and relaxed classroom atmosphere, enabling students to gain more enjoyment from physical activities and thereby improving their overall evaluation and satisfaction with physical education courses (Sánchez-García et al., 2024). Additionally, as another mediating variable, teachers’ emotional support and positive feedback can effectively enhance students’ exercise self-efficacy, and high exercise self-efficacy further enhances students’ physical education class learning satisfaction (Trigueros et al., 2019). Finally, teacher care promotes the improvement of learning satisfaction in physical education classes by enhancing students’ enjoyment of physical activity and exercise self-efficacy, forming a chain of mediating effects among the four variables. Research also shows that teacher care not only directly influences learning satisfaction in physical education classes but also indirectly affects learning satisfaction through the chain of effects “exercise enjoyment → exercise self-efficacy” (Salles et al., 2020; Morales-Sánchez et al., 2021). In summary, teacher care creates a positive learning environment for students, enhances their enjoyment of physical activity, and the positive experience further strengthens their exercise self-efficacy, ultimately contributing to an increase in learning satisfaction in physical education classes.

## 6 Practical significance

This study takes teacher care, exercise enjoyment, and exercise self-efficacy as core variables, systematically exploring the chain-mediated effect of teacher care on sports class learning satisfaction through exercise enjoyment and exercise self-efficacy. This research holds significant practical implications. First, the research findings provide a theoretical basis for physical education teaching reform in higher education institutions, emphasizing the critical role of teacher care in enhancing students’ satisfaction with physical education course learning. This suggests that physical education teachers in higher education institutions should focus on emotional support and positive feedback to create a caring classroom atmosphere. Second, the study reveals the mediating role of exercise enjoyment and exercise self-efficacy in enhancing satisfaction with physical education course learning, providing practical guidance for physical education course design and teaching method innovation, and promoting the implementation of student-centered physical education concepts. Finally, this study provides scientific references for promoting college students’ physical and mental health, stimulating intrinsic motivation for physical education learning, and enhancing the educational effectiveness of physical education courses, thereby playing a positive role in promoting the high-quality development of physical education in higher education institutions.

## 7 Limitations and future directions

This study systematically examined the associations among teacher care, exercise enjoyment, exercise self-efficacy, and college students’ satisfaction with physical education courses. The findings suggest that higher levels of perceived teacher care are associated with greater satisfaction, partially through the sequential mediating roles of exercise enjoyment and exercise self-efficacy. These results enrich the theoretical understanding of student motivation and satisfaction in physical education and provide practical insights for improving teaching quality and promoting students’ physical and psychological development. The study highlights the importance of teachers’ emotional support, classroom climate, and students’ psychological experiences in enhancing learning satisfaction, offering valuable implications for the optimization of physical education courses in higher education.

However, several limitations should be acknowledged. First, the data were collected through a cross-sectional, self-report survey administered during a single classroom session, which may increase the risk of common method bias and limit causal inference. Second, the study sample was drawn from a limited number of universities, which may restrict the generalizability of the findings. Third, the reliance on self-reported data could introduce social desirability bias and subjective response tendencies. Future research could employ longitudinal or experimental designs, use multiple data sources (e.g., teacher evaluations, behavioral observations, or peer reports), and include participants from diverse institutions to validate and extend the present findings. These approaches would strengthen the robustness, ecological validity, and explanatory power of future studies in this field.

## 8 Conclusion

This study systematically revealed the significant promotional effect of teacher care on college students’ physical education class learning satisfaction in Chinese universities, and clarified the chain-like mediating mechanism played by the exercise enjoyment and exercise self-efficacy. The results showed that teacher care not only directly improves students’ physical education class learning satisfaction, but also indirectly promotes satisfaction by enhancing the exercise enjoyment and exercise self-efficacy, thereby enriching the theoretical system of emotional support and psychological mechanisms in the field of physical education. The study emphasizes that teachers should focus on emotional care and positive feedback in physical education classrooms to create a supportive atmosphere, thereby stimulating students’ positive emotional experiences and exercise confidence, and achieving the educational objectives of physical education courses. Although this study has certain limitations in terms of sample size and research methods, its findings provide important theoretical basis and practical references for physical education teaching reforms, course design innovations, and the physical and mental health development of college students. Future research is recommended to combine longitudinal tracking and multi-source data collection methods to further explore the dynamic relationships and underlying mechanisms between teacher care, exercise enjoyment, exercise self-efficacy, and physical education class learning satisfaction, thereby enhancing the scientific rigor and practical value of the research.

## Data Availability

The raw data supporting the conclusions of this article will be made available by the authors, without undue reservation.
