# Supplementary material for: The relationship between teacher care and Chinese college students’ physical education class learning satisfaction: the chain mediation effect of exercise enjoyment and exercise self-efficacy
Source: Front Psychol. 2025 Dec 15;16:1691027. doi: 10.3389/fpsyg.2025.1691027 (PMC12745475; doi:10.3389/fpsyg.2025.1691027)
Supplement: Supplementary file 1 [file Table_1.DOCX]

**X: Teacher Care Scale**

The scale uses a five-point Likert scale for responses and scoring. Respondents select options based on their actual circumstances according to the degree of agreement. Scoring is assigned as follows: “Completely agree” receives 5 points, “Basically agree” receives 4 points, “Possibly agree” receives 3 points, “Basically disagree” receives 2 points, and “Completely disagree” receives 1 point. Scores are summed.

1. If an exercise is too difficult for me, the teacher will provide an alternative exercise or simplify the exercise.

2. During classroom games or presentations, the teacher does not encourage us.

3. The teacher implements safety measures during skill practice in the classroom.

4. The teacher greets us when encountering us outside of class.

5. The teacher pays close attention to me.

6. The teacher provides me with support when conditions permit.

7. The teacher provides me with opportunities to present in class.

8. If I am unwell or sick, the teacher will continue to monitor my recovery.

9. I like my physical education teacher.

10. The teacher uses humorous explanations to stimulate our interest in physical education.

11. The teacher makes me feel important.

12. The teacher analyzes my physical education performance with me and guides me on how to improve.

13. The teacher patiently listens to me and communicates with me.

14. If I don't understand how to complete an exercise, the teacher will patiently explain it to me.

15. The teacher is unfair to me.

16. The teacher encourages us to set our own learning goals.

17. The teacher is friendly to us in class.

18. The teacher does not proactively inquire about my extracurricular physical education activities.

19. During physical fitness exercises, the teacher adjusts the intensity of the exercises appropriately based on our physical condition.

20. The teacher makes me feel outstanding in class.

21. The teacher designs teamwork exercises to encourage us to help each other.

22. The teacher incorporates some health and wellness knowledge into physical education theory classes and encourages us to develop healthy lifestyles.

23. My relationship with the physical education teacher is both teacher and friend.

**M1: “Physical Activity Enjoyment Scale”**

5 = strongly agree, 4 = agree, 3 = unsure, 2 = somewhat disagree, 1 = strongly disagree

1. I can enjoy the fun of it

2. I feel bored

3. I don't like physical activities

4. I feel very happy

5. Physical activities have no fun at all

6. Physical activities give me energy

7. Physical activities make me feel discouraged

8. Physical activities are very enjoyable

9. My body feels good during the activity

10. I gain something from physical activities

11. Physical activities are very exciting

12. Physical activities frustrate me

13. Physical activities are not enjoyable at all

14. Physical activities give me a strong sense of accomplishment

15. Physical activities make me feel good

16. I seem to prefer doing other things

**M2: Exercise Self-Efficacy Scale**

1 = Strongly disagree, 2 = Disagree, 3 = Undecided, 4 = Agree, 5 = Strongly agree

1. I exercise most days of the week.

2. I can ask my parents or other adults to exercise with me.

3. Even if I can watch TV or play video games, I can still exercise during my free time most days of the week. 4. Even if it is very hot or cold outside, I can still exercise on most days of the week.

5. I can ask my best friend to exercise with me on most days of the week.

6. Even at home, I can still exercise.

7. I can exercise because I know how to exercise.

8. No matter how busy I am, I can still exercise during my free time on most days of the week.

**Y: Physical Education Class Learning Satisfaction Scale**

This scale is divided into five dimensions: 1 = completely disagree, 2 = somewhat disagree, 3 = neutral, 4 = agree, 5 = strongly agree.

1. Learning is flexible and autonomous.

2. The relationship between teachers and students is harmonious.

3. Appropriate physical activity levels

4. Appropriate teaching methods

5. Rich and diverse teaching content

6. Good teaching organization and learning atmosphere

7. Teachers with good moral character

8. Teachers demonstrate good professional ethics and conduct

9. Teachers possess extensive professional knowledge and skilled expertise

10. Teachers exhibit a serious teaching attitude 1

11. Teachers can accurately identify teaching priorities

12. Teachers communicate clearly 1

13. Teachers maintain an appropriate pace in their teaching

14. Teachers foster friendships among students

15. Teachers receive encouragement and support from students

16. Competition and cooperation in teaching competitions are good

17. Students can share sports equipment and facilities

18. Relationships among students are good

19. Interaction among students is good

20. The hygiene of sports facilities is good

21. The safety of sports equipment is good

22. The quantity of sports equipment is adequate

23. The quality of sports equipment is good

24. Teacher grading is fair

25. The exam format is appropriate

26. The grading criteria for exams are reasonable

27. The structure of the grading system is reasonable
